# O025: Organisational transformation – the application of novel change techniques & social media understanding to motivate infection preventionists

**DOI:** 10.1186/2047-2994-2-S1-O25

**Published:** 2013-06-20

**Authors:** J Storr, H Loveday, L Wharton, D Flaxman, D Wright, E Curran, M Tannahil, G Thirkell, N Wiggleworth, P Cattini, J Wilson, C Kilpatrick

**Affiliations:** 1 Infection Prevention Society, West Lothian, UK; 2 IPS, IPS, London, UK

## Introduction

The Infection Prevention Society (IPS) is a charity that supports members to ensure no person is harmed by a preventable infection. In 2009, IPS began a strategy planning and evaluation exercise that has acted as the catalyst for transformation, achieved through the use of innovative change techniques and a social media campaign.

## Objectives

To establish a new strategy and social media campaign.

## Methods

Qualitative and quantitative approacheswere used; a needs assessment of members, a creative thinking workshop using 3 novel techniques drafted a vision, mission, strategic aims and objectives, feedback exercises followed at IPS' annual conferences. This included a Strategy Decision Tree and Strategy Wall to gather free text that was analysed. In parallel, a baseline survey of members’ use of social media was issued and a follow up process for twitter analysis established.

## Results

In 2011, IPS issued strategic objectives: a) to lead, shape and inform the infection prevention agenda; b) to generate and promote the evidence base for infection prevention, and c) to be the organisation of choice to sustain improvement.

The member survey found that equal proportions of members (39%) thought that use of Twitter would enhance and not enhance IPS. Implementation and on-going evaluation exercises were designed from this intelligence. In 2011, 1% of members stated that Twitter would serve a purpose. In 2013, 37% of IPS members were signed up to Twitter. Within 1 month the impact of the @IPS_infection Twitter account increased 2 and a half fold, (reach - 8,047 to 27,146 and impressions - 17,502 to 44,529 respectively).

## Conclusion

This approach engaged members in strategy development and allowed for articulation of achievable but stretching objectives. The use of social media and Twitter in particular is being used to reinvigorate networking and influence, and is already yielding results. A range of measures are being employed to support on-going strategy implementation, to track progress and to role model to other societies.

## Disclosure of interest

None declared.

